# Sheng-Mai-Yin inhibits doxorubicin-induced ferroptosis and cardiotoxicity through regulation of Hmox1

**DOI:** 10.18632/aging.205062

**Published:** 2023-09-28

**Authors:** Peina Meng, Zhaoyang Chen, Tianhui Sun, Lili Wu, Yifan Wang, Tianwei Guo, Jin Yang, Jiebin Zhu

**Affiliations:** 1 Department of Preventive Medicine, Changshu Hospital Affiliated to Nanjing University of Chinese Medicine, Changshu, China; 2 School of Traditional Chinese Medicine and School of Integrated Chinese and Western Medicine, Nanjing University of Chinese Medicine, Nanjing, China; 3 School of Pharmacy, Nanjing University of Chinese Medicine, Nanjing, China; 4 Department of Cardiology, Nanjing First Hospital, Nanjing Medical University, Nanjing, China

**Keywords:** Sheng-Mai-Yin, doxorubicin, Hmox1, ferroptosis, cardiotoxicity

## Abstract

Doxorubicin (DOX) is a potent chemotherapeutic drug used for treating various cancers. However, its clinical use is limited due to its severe cardiotoxicity, which often results in high mortality rates. Sheng-Mai-Yin (SMY), a Traditional Chinese medicine (TCM) prescription, has been reported to exert a cardioprotective effect in various cardiovascular diseases, including DOX-induced cardiotoxicity (DIC). This study aimed to provide novel insights into the underlying cardioprotective mechanism of SMY. SMY, composed of Codonopsis pilosula (Franch.), Ophiopogon japonicus (Thunb.), and Schisandra chinensis (Turcz.) at a ratio of 3:2:1, was intragastrically administered to male C57BL/6 mice for five days prior to the intraperitoneal injection of mitoTEMPO. One day later, DOX was intraperitoneally injected. Hematoxylin-eosin staining and Sirius red staining were carried out to estimate the pharmacological effect of SMY on cardiotoxicity. Mitochondrial function and ferroptosis biomarkers were also examined. AAV was utilized to overexpress Hmox1 to confirm whether Hmox1-mediated ferroptosis is associated with the cardioprotective effect of SMY on DOX-induced cardiotoxicity. The findings revealed that SMY therapy reduced the number of damaged cardiomyocytes. SMY therapy also reversed the inductions of cardiac MDA, serum MDA, LDH, and CK-MB contents, which dramatically decreased nonheme iron levels. In the meantime, SMY corrected the changes to ferroptosis indices brought on by DOX stimulation. Additionally, Hmox1 overexpression prevented SMY's ability to reverse cardiotoxicity. Our results showed that SMY effectively restrained lipid oxidation, reduced iron overload, and inhibited DOX-induced ferroptosis and cardiotoxicity, possibly via the mediation of Hmox1.

## INTRODUCTION

As a valuable chemotherapeutic drug, doxorubicin (DOX) is used to treat various types of cancers [1]. Whereas, the clinical application of doxorubicin is limited due to its cardiotoxic effects including refractory degenerative cardiomyopathy and even irreversible heart failure [2]. The separate phase clinical trials in phase III revealed that 26% of cancer patients who received doxorubicin treatment with an accumulated dose of 550 mg/m^2^ developed irreversible congestive heart failure [3]. In addition, there was a high cardiotoxicity risk among patients with lymphoma who received DOX treatment [4]. Therefore, there is an immediate need for the exploration of therapeutic strategies aiming at reducing doxorubicin-induced cardiotoxicity (DIC) without compromising its therapeutic function.

In recent years, Traditional Chinese medicine (TCM) has gradually become more popular for treating difficult and complicated diseases. Sheng-Mai-Yin (SMY), a classic prescription from TCM, was first documented in Qian Jin Yao Fang, the ancient prescriptions of emergencies, which were written 1300 years ago [5]. SMY is composed of Codonopsis pilosula (Franch.), Ophiopogon japonicus (Thunb.), and Schisandra chinensis (Turcz.). All the plant names have been checked with MPNS (http://mpns.kew.org) on Dec 28th, 2022. The three plants used in SMY have a long history in traditional local medicine in Asian countries like China, Korea, and Japan [6–8]. According to TCM theory, each plant that makes up SMY has its role. Codonopsis pilosula replenishes Qi and promotes body fluid production, playing the role of sovereign. Radix Ophiopogonis is used as a minister to nourish Yin and remove heat. Schisandra chinensis is used to stop sweat and produce more body fluid as an assistant. Hence, the absence of any one of the three plants will lead to a reduction in the curative effect of SMY. Clinically, SMY is often used to supplement Qi and nourish Yin in TCM theories. Since the lack of Qi and Yin is a common phenomenon in cancer patients who receive radio or chemical therapy, the treatment of SMY in these patients has achieved well effects and has become a research hotspot [9, 10]. Moreover, SMY was also widely applied for the interventions of various cardiovascular disorders including myocarditis [11], diabetic cardiomyopathy [12], myocardial infarction [13], myocardial ischemia-reperfusion [14], and heart failure [15]. These cardioprotective effects were related to the anti-ischemia, anti-hypoxia, and anti-oxidative activities [16–18]. Several articles reported that SMY relieved cardiomyocyte injury induced by DOX [19–21], partially through the activation of the Nrf2/Keap1 signaling pathway and resistance to oxidative stress [22]. Nrf2 was also reported to be a potential target for treating DIC [23]. Interestingly, the excessive activation of Nrf2 might induce ferroptosis of cardiomyocytes due to overexpression of Hmox1 [24]. Herein, we aimed to elucidate the mechanism of the cardioprotective effects of SMY in doxorubicin-caused ferroptosis and cardiotoxicity based on Hmox1 in the present study.

Ferroptosis, the novel regulated cell death (RCD) form, is featured by the accumulation of iron, reactive oxygen species (ROS), and iron-related lethal lipid peroxides [25]. Hmox1 (Heme oxygenase-1) is an inducible enzyme that elevates after oxidative stress and catalyzes cellular hemoglobin to produce free ferrous iron, carbon monoxide, and biliverdin [26]. Hmox1 is traditionally seen as an antioxidative enzyme because it degrades pro-oxidant free heme to antioxidant biliverdin [27]. However, the ferrous iron accumulated from the Hmox1 reaction is an abundant source to trigger ferroptosis in various cardiovascular diseases including DOX-stimulated cardiomyopathy [28, 29]. Hmox1 is therefore deeply involved in iron metabolism and participated in DOX-induced mitochondrial dysfunction, ferroptosis, and cardiomyocyte injury [30].

The present study aimed to shed new light on the fundamental mechanism of the cardioprotective effect of SMY and propose the TCM solution for attenuating doxorubicin-induced cardiotoxicity. This study was conducted to estimate the protective mechanism of SMY in doxorubicin induced cardiotoxicity by investigating Hmox1 expression, mitochondrial iron overload, and lipid peroxidation.

## RESULTS

### Chemical profiling for Sheng-Mai-Yin

**Figure 1:**
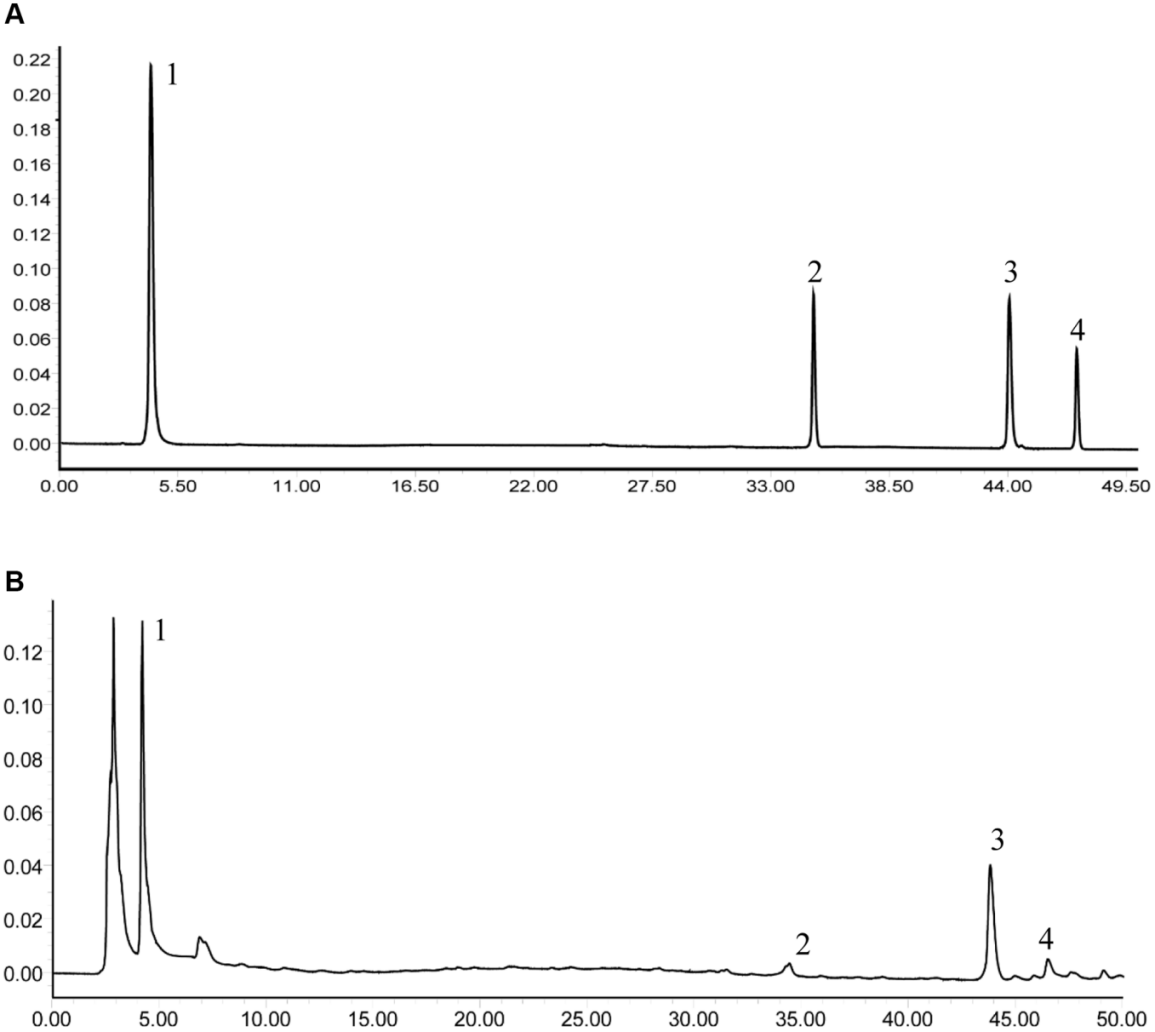
**The HPLC analysis of SMY and its main components, including 5-HMF (1), lobetyolin (2), Schisandrin B (3), schizandrin (4).** (**A**) Standards. (**B**) Samples.

The main components of SMY were 5-hydroxymethyl-furfura (5-HMF), lobetyolin, Schisandrin B, schizandrin. As shown in Figure 1, the comparative analyses of the molecular retention times of the standards and SMY samples were as follows: 5-HMF 14.08 mg/g, lobetyolin 0.52 mg/g, Schisandrin B 1.27 mg/g, schizandrin 0.60 mg/g.

### Sheng-Mai-Yin prevented DOX-induced cardiac injury

**Figure 2:**
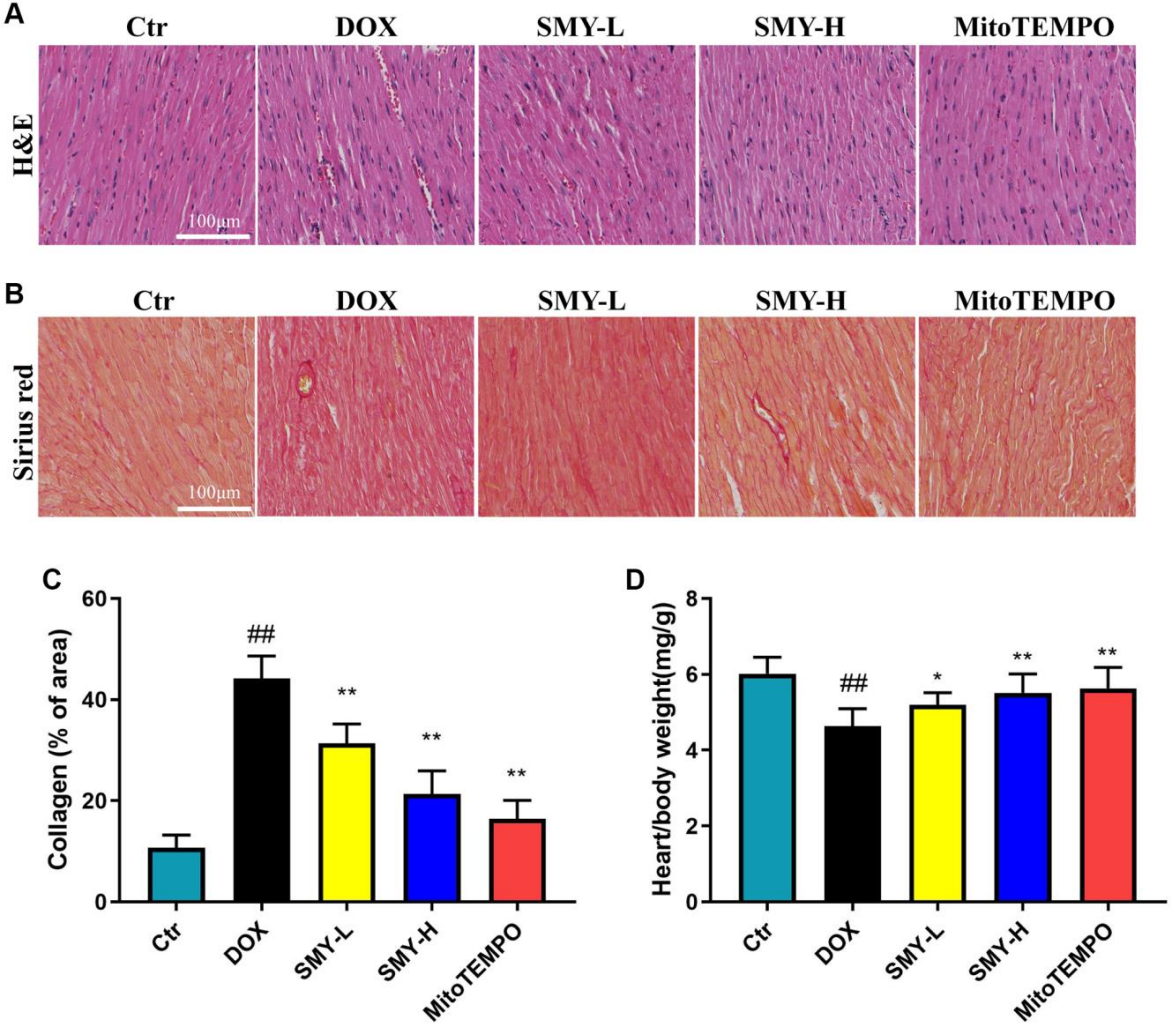
**SMY prevented DOX-induced cardiomyopathy.** (**A**, **B**) Cardiac sections were prepared from control mice and mice treated with DOX. SMY-L or SMY-H or mitoTEMPO were treated. The heart tissues were stained with hematoxylin and eosin (H&E, Top) or Sirius red (Bottom). (**C**) Cardiac collagen quantification (% of collagen of area) in heart tissue. (**D**) The heart/body weight ratio was calculated in control mice and mice treated with DOX with or without SMY-L or SMY-H or mitoTEMPO (*n* = 9 mice per group). The results were presented as mean ± SEM. ^##^means compared with control group, *P* < 0.01; ^*^means compared with DOX group, *P* < 0.05, ^**^means compared with DOX group, *P* < 0.01.

To verify the effects of SMY on DOX-induced cardiac injury, HE staining and Sirius red staining was used for histopathological evaluation. As shown in Figure 2A, the morphology and structure of cardiomyocytes were intact in the control group, while the cardiomyocytes in the DOX group presented a necrotic and irregular arrangement. However, the SMY treatment improved the myocardial damage caused by DOX, which is similar to the effects of mitoTEMPO, a mitochondrial-targeted antioxidant. Sirius red staining revealed that SMY or mitoTEMPO inhibits the excessive deposition of extracellular matrix collagen in heart slices (Figure 2B, 2C). The ratio of heart to body weight of mice was decreased by DOX treatment, indicating the heart damage caused by DOX. Interestingly, SMY or mitoTEMPO effectively restores the ratio (Figure 2D) Moreover, echocardiography has revealed that SMY administration improved cardiac function in DOX treated mice (Supplementary Figure 1).

### The effects of Sheng-Mai-Yin on iron overload, lipid peroxidation and serum enzymology

**Figure 3:**
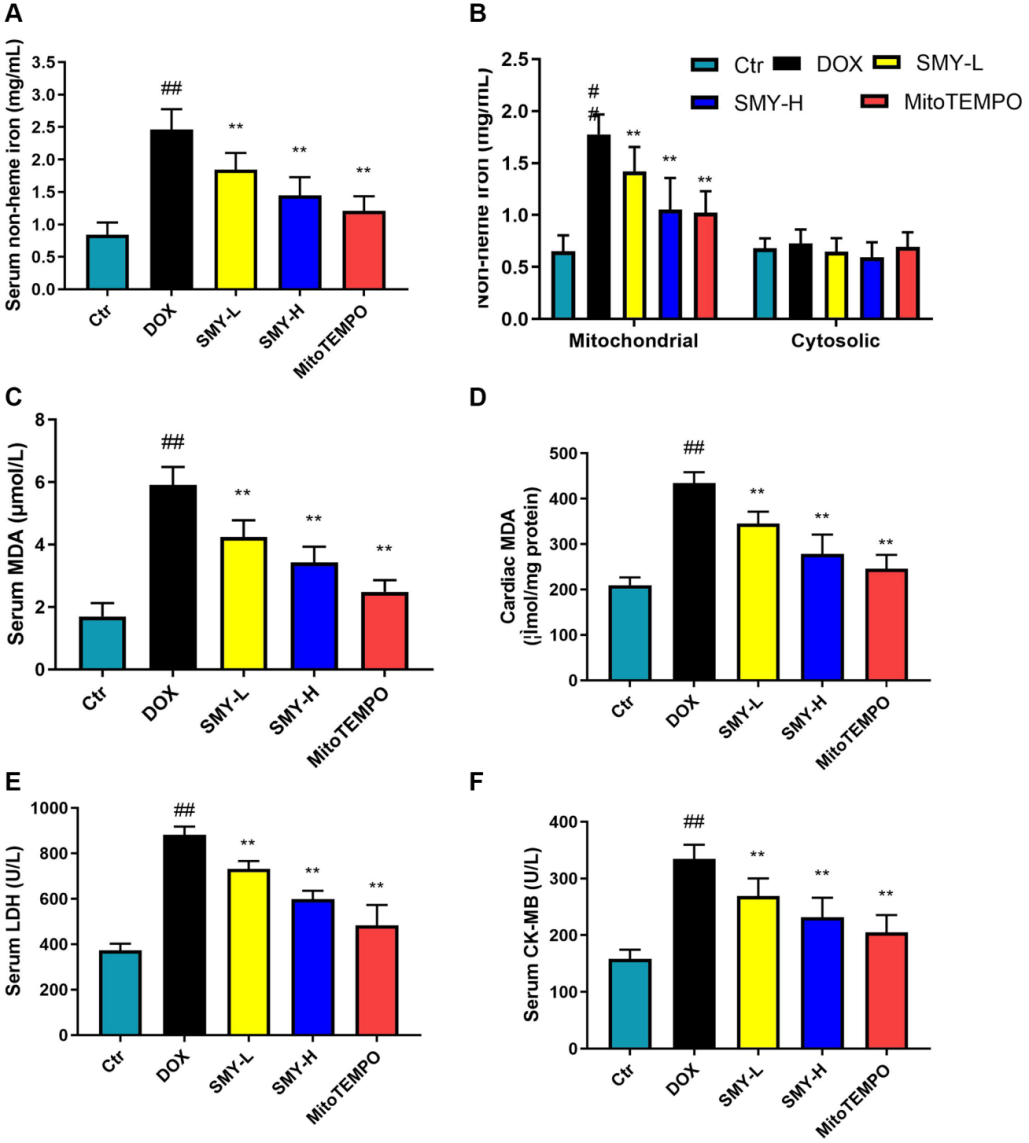
**SMY inhibited iron overload, lipid peroxidation, and serum enzymology.** (**A**, **B**) Serum (**A**), cardiac mitochondrial, and cytosolic (**B**) nonheme iron were detected by an iron assay kit (#ab83366, Abcam). (**C**, **D**) Serum (**C**) and cardiac (**D**) MDA contents were measured by lipid peroxidation MDA assay kit (Beyotime, S0131S) in control mice and mice treated with DOX with or without SMY or mitoTEMPO. (**E**, **F**) Serum LDH (**E**) and CK-MB (**F**) levels were examined by commercial assay kits (Beyotime, C0016 and ZCIBIO, ZC-38269) according to the manufacturer’s instructions. The results were presented as mean ± SEM. ^##^means compared with control group, *P* < 0.01; ^*^means compared with DOX group, *P* < 0.05, ^**^means compared with DOX group, *P* < 0.01.

The mitochondrial lipid peroxidation induced by mitochondrial iron accumulation is a crucial source of cardiac ferroptosis induced by DOX [24]. As shown in Figure 3A, 3B, serum and mitochondrial nonheme iron levels were substantially elevated by DOX treatment compared with the control group, while SMY treatment significantly reduced the nonheme iron levels. Additionally, the content of cardiac MDA, the levels of serum MDA, LDH and CK-MB were increased with the treatment of DOX (Figure 3C–3F). On the contrary, the inductions of cardiac MDA, serum MDA, LDH, and CK-MB contents were reversed by SMY treatment.

### Sheng-Mai-Yin scavenged ROS generation and alleviated mitochondria dysfunction

**Figure 4:**
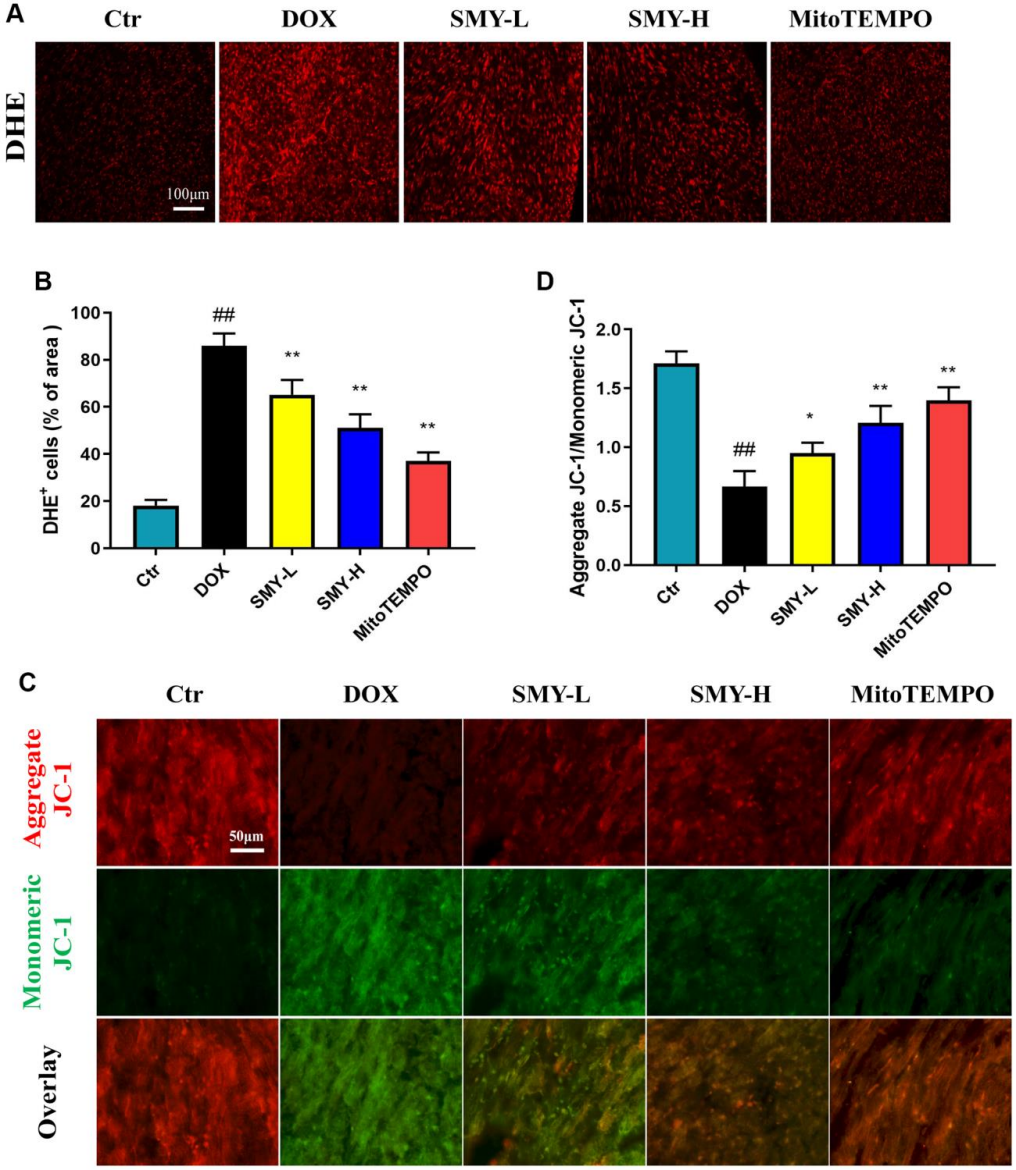
**SMY scavenged ROS generation and alleviated mitochondria dysfunction.** (**A**) Images of DHE staining of ROS. (**B**) Histogram showed the DHE+ cells of the area in different groups. (**C**) Representative images of JC-1 staining in different groups. Red fluorescence represented aggregation of JC-1, and green fluorescence represented monomeric JC-1. (**D**) Histogram of aggregate JC-1/monomeric JC-1. The JC-1 probe was applied to measure ΔΨm, which was expressed as the ratio of red (the aggregate form of JC-1, which denotes intact ΔΨm) to green (a monomeric form of JC-1, which signifies dissipation of ΔΨm). The results were presented as mean ± SEM. ^##^means compared with control group, *P* < 0.01; ^*^means compared with DOX group, *P* < 0.05, ^**^means compared with DOX group, *P* < 0.01.

Reactive oxygen species (ROS) production led to oxidative stress which contributes to the cardiac damage caused by DOX [31]. As vividly depicted in Figure 4A, 4B, the treatment of DOX triggered a robust generation of ROS, which was reduced with the treatment of SMY and mitoTEMPO.

To further examine the source of ROS, we measured the mitochondrial function by mitochondrial membrane potential (ΔΨm) with JC-1 staining. It was found that DOX group had significantly more monomers compared with the control group which had more aggregates. However, SMY treatment remarkably decreased the ratio of aggregates JC-1/monomers JC-1 (Figure 4C, 4D) Furthermore, SMY treatment successfully restored the damaged mitochondrial morphology, as observed through transmission electron microscopy (Supplementary Figure 2).

### The effect of Sheng-Mai-Yin on ferroptosis

**Figure 5:**
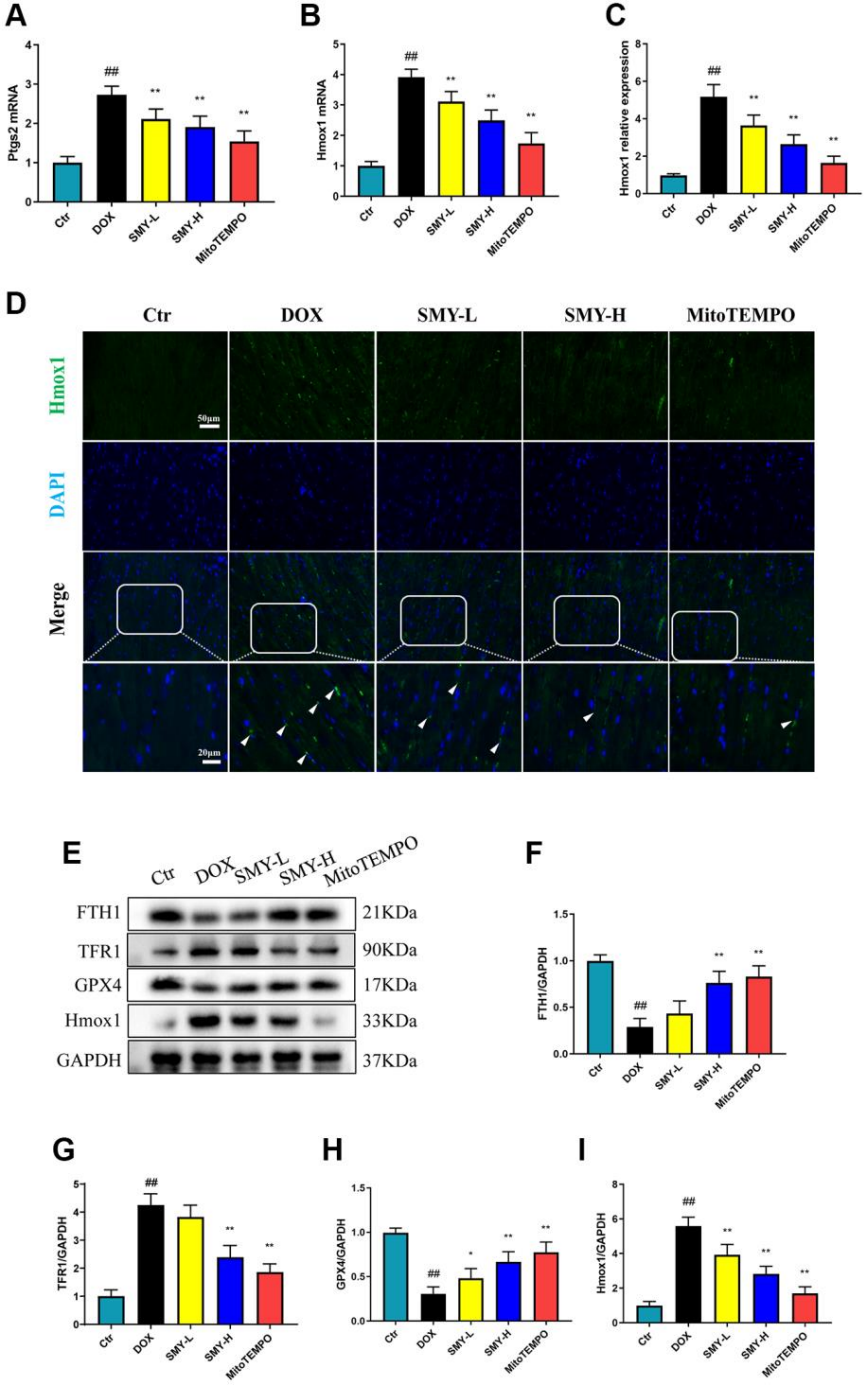
**The effect of SMY on molecular markers of ferroptosis.** (**A**, **B**) Ptgs2 mRNA (**A**) and Hmox1 mRNA (**B**) levels were measured by qPCR. (**C**) Quantification of the intensity of immunofluorescence of Hmox1. (**D**) Representative images of immunofluorescence of Hmox1. The green fluorescence represented Hmox1 and the blue fluorescence indicated cell nucleus. (**E**) Representative blot images of FTH1, TFR1, GPX4, and Hmox1 protein levels. (**F**) Analysis of FTH1 protein levels by column graph, adjusted by GAPDH. (**G**) Analysis of TFR1 protein levels by column graph, adjusted by GAPDH. (**H**) Analysis of GPX4 protein levels by column graph, adjusted by GAPDH. (**I**) Analysis of Hmox1 protein levels by column graph, adjusted by GAPDH. The results were presented as mean ± SEM. ^##^means compared with control group, *P* < 0.01; ^*^means compared with DOX group, *P* < 0.05, ^**^means compared with DOX group, *P* < 0.01.

Ptgs2, a gene encoding cyclooxygenase-2 (COX-2), is a putative molecular marker of ferroptosis [32]. We found that after 4 days of doxorubicin treatment, the DOX group exhibits almost threefold of Ptgs2 mRNA expression compared with that of the control group, and SMY treatment effectively reduced the Ptgs2 mRNA levels (Figure 5A). Moreover, DOX treatment resulted in elevation in Hmox1 mRNA levels (Figure 5B) and immunofluorescence intensity (Figure 5C, 5D), which was relevant to the nuclear translocation of Nrf2. Our results depicted that SMY treatment also inhibited the expression of Hmox1 (Figure 5B–5D). To further research the effect of SMY on ferroptosis, we detected the ferroptosis biomarkers, including FTH1, TFR1, GPX4, GSH and Hmox1 levels. As shown in Figure 5E–5I and Supplementary Figure 3, SMY successfully reversed the alterations of these indices caused by the stimulation of DOX.

### Overexpression of Hmox1 canceled the effect of Sheng-Mai-Yin on ferroptosis biomarkers

**Figure 6:**
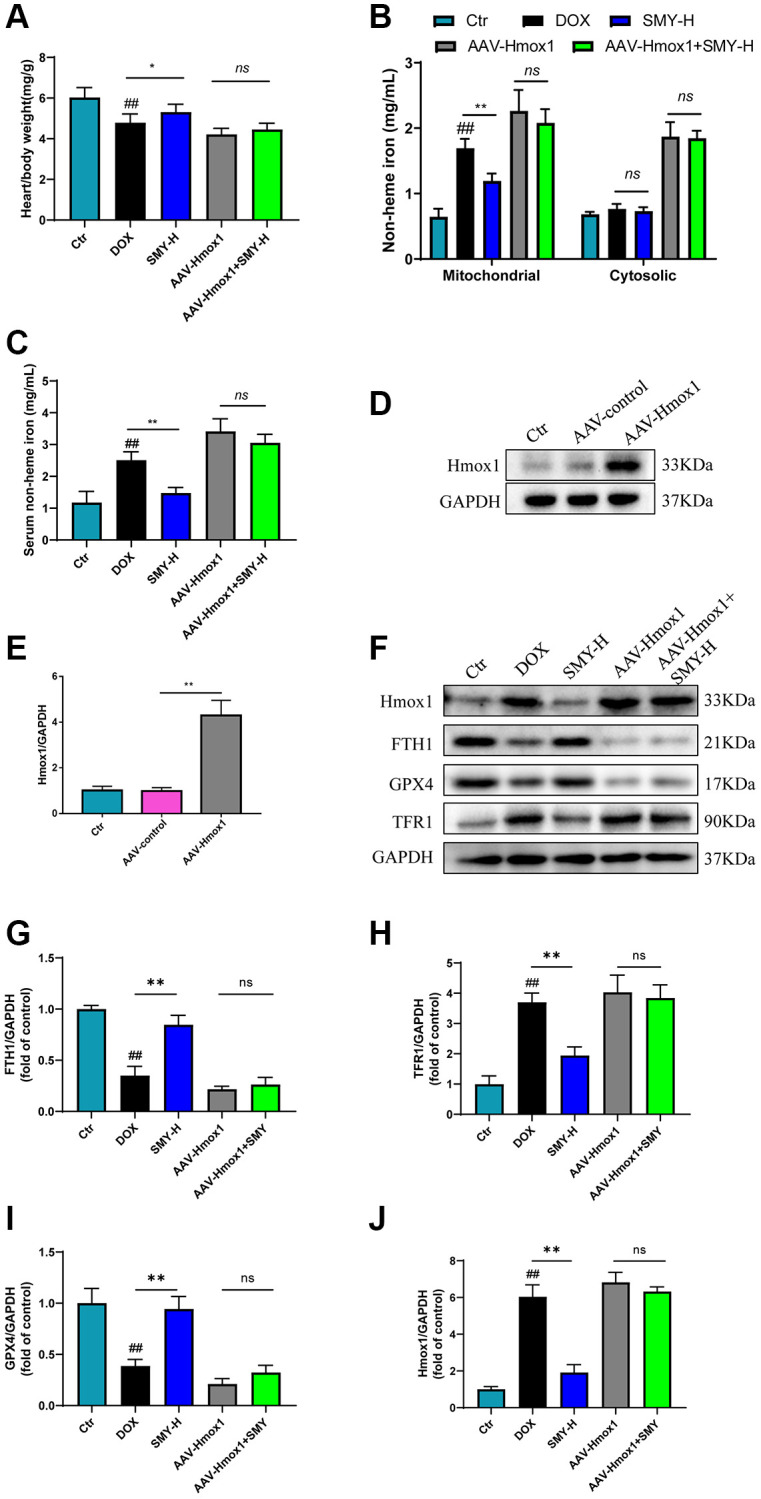
**Overexpression of Hmox1 counteracted the effect of SMY on ferroptosis.** (**A**) The heart/body weight ratio was calculated. (**B**, **C**) Cardiac mitochondria and cytosolic nonheme iron (**B**) and serum nonheme iron (**C**) were detected by an iron assay kit (#ab83366, Abcam) in mice treated with DOX with or without SMY or Hmox1-OE. (**D**, **E**) Western blot analysis of Hmox1 and GAPDH protein levels in control mice, AAV-control mice, and AAC-Hmox1 mice. (**F**–**J**) Western blot analysis of FTH1, TFR1, GPX4, and Hmox1 protein contents. The results were presented as mean ± SEM. ^*^means compared with the other group, *P* < 0.05, ^**^means compared with the other group, *P* < 0.01.

To verify whether Hmox1 played a key role in the effect of Sheng-Mai-Yin on DOX-induced cardiotoxicity, we overexpressed the gene Hmox1 in mice with the adeno-associated virus. The same protein levels of Hmox1 were observed in both control and AAV-control mice, indicating that AAV transfection had no influence on Hmox1 expression. As expected, the expression of Hmox1 in the AAV-Hmox1 group was prominently elevated compared with those of other groups (Figure 6D, 6E). As illustrated in Figure 6A, the ratio of the heart/body weight was elevated by SMY-H compared with that of single DOX treatment, while the overexpression of Hmox1 abolished this effect. Similarly, although nonheme iron was decreased by SMY-H+DOX treatment in mitochondrial and serum, the mitochondrial and serum nonheme iron of Hmox1 overexpression mice showed no significant difference between single DOX treatment and SMY-H+DOX treatment (Figure 6B, 6C). Additionally, the effects of SMY on the protein expression of FTH1, TFR1, GPX4, and Hmox1 were overwhelmingly abrogated by the overexpression of Hmox1 (Figure 6F–6J).

### Overexpression of Hmox1 blocked the effect of Sheng-Mai-Yin on Ferroptosis morphologically

**Figure 7:**
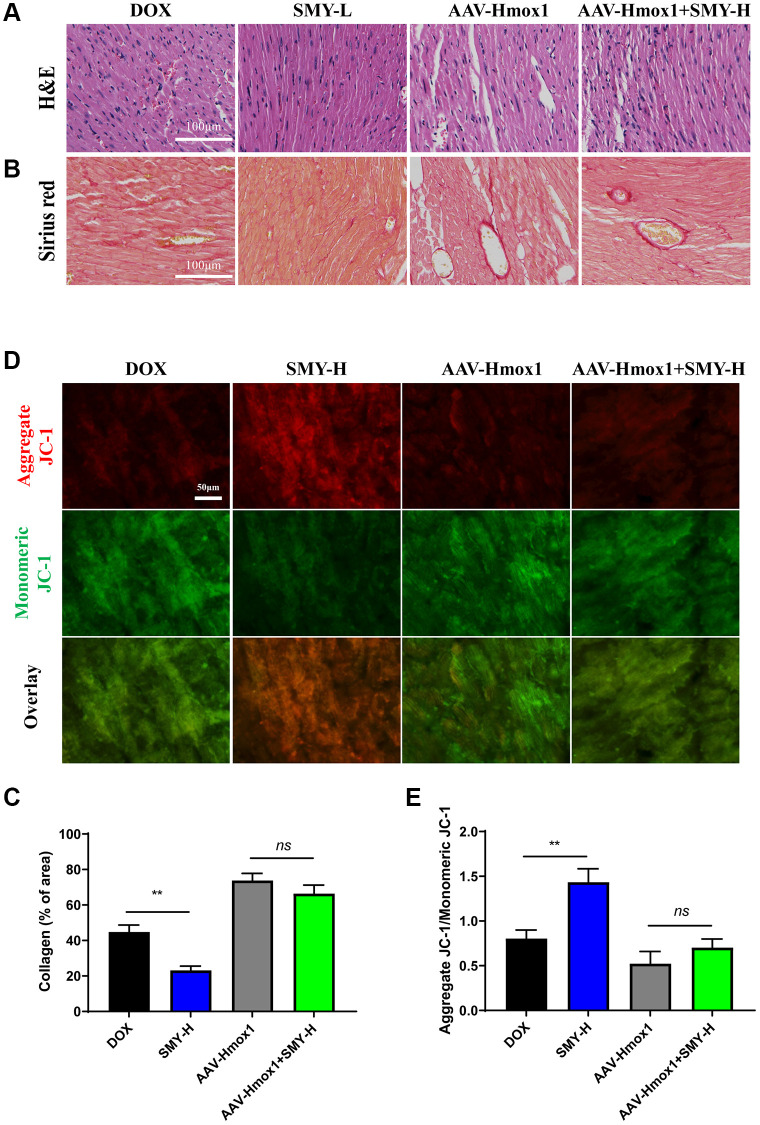
**Overexpression of Hmox1 counteracted the effect of SMY on DIC.** (**A**) Hematoxylin and eosin (H&E) staining of heart tissues. (**B**) Sirius red staining of mice treated with DOX with or without SMY or Hmox1-OE. (**C**) Cardiac collagen quantification (% of collagen of area) of heart tissues. (**D**) Representative images of JC-1 of heart tissues. JC-1 aggregation is represented by red fluorescence and monomeric JC-1 formation by green fluorescence. (**E**) Histogram of aggregate JC-1/ monomeric JC-1 in mice treated with DOX with or without SMY or Hmox1-OE. The results were presented as mean ± SEM. ^*^means compared with the other group, *P* < 0.05, ^**^means compared with the other group, *P* < 0.01.

As depicted in Figure 7A–7C, SMY-H reduced the collagen area compared with that of the DOX group, which disappeared in Hmox1 overexpression mice. We measured the mitochondrial function with JC-1 staining again. As depicted in Figure 7D, 7E, SMY-H increased the ratio of aggregates JC-1/monomers JC-1, which was abolished by the overexpression of Hmox1. Furthermore, we observed that the overexpression of Hmox1 attenuated the protective effects of SMY on cardiac function and mitochondrial morphology, as assessed through echocardiography and transmission electron microscopy (Supplementary Materials).

## DISCUSSION

Numerous studies have been conducted on the fundamental mechanisms of doxorubicin-induced cardiotoxicity (DIC), including oxidative stress injury, necroptosis, pyroptosis, apoptosis and autophagy [33–35]. In addition, a recent study found that ferroptosis controlled cardiotoxicity after the DOX challenge, in which Hmox1 played a pivotal role in elevating iron levels, followed by lipid peroxidation and cell membrane breakage [24]. Heme oxygenase-1 (Hmox1), a rate-limiting enzyme of heme that converts hemoglobin into CO, Fe^2+^, and biliverdin, is regulated by Nrf2 (nuclear factor erythroid 2-related factor 2) [36]. The Nrf2/Hmox1 axis has long been regarded as a cardioprotective signaling pathway that exerts protective effects on DIC by regulating autophagy and oxidative stress [37–39]. However, Fang et al. reported that the upregulated Hmox1 catalyzed the degradation of heme to generate ferrous iron, causing mitochondrial iron overload and increasing ferroptosis [24], which provides a novel mechanism to elucidate the cardioprotective effects of SMY on doxorubicin-caused cardiotoxicity. Our study found that SMY effectively reduced iron overload and lipid oxidation in mitochondria and maintained mitochondria function in the DIC model. SMY successfully reduced interstitial fibrosis of mice heart tissues and serum LDH and CK-MB levels in DOX-treated mice, indicating that SMY attenuated cardiotoxicity caused by DOX.

Nonheme iron accumulation in mitochondrial is one of the typical characteristics of ferroptosis in DOX-treated cardiomyocytes. Clinically, an accumulation of iron inside the cardiac mitochondria was found in patients who received DOX treatment [40, 41]. Our study also validated that both serum and myocardial mitochondrial nonheme irons were elevated post-DOX treatment, while SMY significantly reversed it. The accumulation of nonheme iron is regulated by FTH1 and TFR1. FTH1 (ferritin heavy chain 1) is one of the subunits of ferritin, which serves as a vital molecule for iron storage [42]. TFR1 (transferrin receptor 1) is a cell membrane protein that mediates the uptake of iron, leading to the overload of free intracellular iron [43]. In brief, the downregulation of FTH1 promotes iron storage and upregulates TFR1 expression, which enhances iron uptake and contributes to iron overload, especially in mitochondria. Excessive iron generates abundant reactive oxygen species (ROS) which contributes to lipid peroxidation and results in ferroptosis through the Fenton reaction [44, 45]. Of note, DOX treatment was reported to downregulate GPX4 (glutathione peroxidase 4) and induce an excess of lipid peroxidation, leading to mitochondria-dependent ferroptosis through the DOX-Fe^2+^ complex in mitochondria [46]. As the most prevalent byproduct of lipid peroxidation [47], Malondialdehyde (MDA) was elevated after DOX treatment, which was reversed by SMY. Considerable evidence supported that DOX-induced cardiotoxicity was related to the excessive generation of intracellular ROS, especially in mitochondria [48–50]. Hence, we utilized mitoTEMPO, a mitochondria-targeted antioxidant [51], as a positive drug in the present study. The dosage of 5 mg/kg body weight in C57BL/6 mice was proved to exhibit well effects against DOX-induced cardiotoxicity [24, 52], which was also proved in the present study. Based on the above analyses, it was obvious that the iron overload and lipid oxidation of mitochondria was pivotal for SMY-mediated ferroptosis in DIC.

As previously illustrated, the activation of the Nrf2/Hmox1 pathway might contribute to the cardioprotective effect of SMY on ferroptosis and DIC. Hmox1, an enzyme degrading hemoglobin, was reported to elevate in murine cardiomyocytes after DOX treatment [53], which is consistent with our observation that Hmox1 was increased in DOX-treated mice. Moreover, overexpression of Hmox1 was reported to exacerbate cardiomyocyte injury in DOX-treated mice [54], and inhibition of Hmox1 overexpression was proved to effectively decrease cellular ferrous accumulation and protect against ferroptosis [55]. In addition, a previous study unraveled that the silence of Hmox1 diminished ROS levels and promoted GPX4 expression, which eliminated Hmox1 mediated ferroptosis in diabetic atherosclerosis [56]. Furthermore, Qian et al. reported that knockout of Hmox1 reduced ROS contents, improved mitochondrial membrane potential dysfunction, decreased Fe^2+^ concentrations, and elevated the levels of GSH and GPX4 in DOX-treated HL-1 cells [30]. Taken together, it’s easy to see that Hmox1 plays a crucial role in iron metabolism and properly regulates DOX-induced cardiovascular disease, which is consistent with our research that overexpression of Hmox1 significantly canceled the cardioprotective effects of SMY in DOX-treated mice.

Since SMY is composed of three TCM plants (Codonopsis pilosula, Radix Ophiopogonis, and Schisandra chinensis) which have large amounts of chemical compounds, the compounds in SMY responsible for its cardiovascular protective effects against DOX-induced cardiotoxicity have been variously reported. Codonopsis pilosula was reported to attenuate AngII plus Leu^27^-IGFII-induced calcium influx and apoptosis in H9c2 cardiomyocytes [57], but the responding effective compound in Codonopsis pilosula is still elusive. Ophiopogonin D, a steroidal saponin isolated from Radix Ophiopogonis, alleviated the DOX-induced autophagy by decreasing the LC3-II/LC3-I ratio and downregulating the expression of both phosphorylated c-Jun N-terminal kinase and extracellular signal-regulated kinase in H9c2 cells [58]. Schisandrin B, a dibenzocyclooctadiene derivative isolated from the fruit of Schisandra chinensis, was reported to alleviate DOX-induced cardiotoxicity via antioxidative and anti-inflammatory effects [59]. Based on the above research, we have more confidence in believing that the combined use of all three plants in SMY will have better effects against DOX-induced cardiotoxicity.

In conclusion, our study illustrated the cardioprotective effect of SMY on DOX-induced cardiotoxicity. The possible mechanism relied on Hmox1-mediated ferroptosis. Our work enriched the mechanism of the cardioprotective effects of SMY on doxorubicin-induced ferroptosis and cardiotoxicity and laid a solid foundation for promoting the clinical application of SMY in various cardiovascular diseases including adverse effects of chemotherapy.

## MATERIALS AND METHODS

### Antibodies and reagents

Antibodies including Ferritin Heavy Chain (FTH1) antibody (ab183781), Transferrin Receptor 1 (TFR1) antibody (ab269513), Glutathione Peroxidase 4 (GPX4) antibody (ab125066), and Heme Oxygenase 1 (Hmox1) antibody (ab52947) were purchased from Abcam (Cambridge, UK). Antibodies including GAPDH (#5174), mouse IgG (H+L) (#14709), and rabbit IgG (H+L) (#14708) were obtained from CST (NY, USA). MitoTEMPO (#SML0737) and doxorubicin (#D807083) were purchased from Sigma (Darmstadt, Germany).

### Preparation and assessment of Sheng-Mai-Yin

300 grams of Codonopsis pilosula, 100 grams of Fructus Schisandrae, and 200 grams of Radix Ophiopogonis were put in a flask together, and immersed in 10 times the amount of water. The sample was cooked for 2 h the first time, then 1.5 h at the second time. The decoction was combined, and filtered. The filtrate was concentrated under reduced pressure into extract (30.2 g). The chemical profile of SMY was analyzed by high-performance liquid chromatography (HPLC).

### Animals and experimental protocol

8 weeks old Male C57BL/6 mice weighing 18-22 grams each were purchased from SiPeiFu (Beijing, China) Biotechnology Co., Ltd. License: SCXK (Beijing, China) 2019-0010. All mice were fed and housed in a standard environment with a temperature of 22 ± 2°C, a dark/light cycle of 12/12 h and a humidity of 40–60%, and ad libitum access to a chow diet and water. Animal care was performed according to the guidelines of the experimental institute of Nanjing University of Chinese Medicine. All animal experiments were approved by the Medical Ethics Committee of University (202205A091).

The mice were randomly assigned to the control group, DOX (10 mg/kg/day) group, SMY-L (135 mg/kg/day) group, SMY-H (270 mg/kg/day) group, and mitoTEMPO (5 mg/kg/day) group, with 9 animals per group. After a week of adaptive feeding, the mice of control group, DOX group, SMY-L group and SMY-H group were intragastrically treated with distilled water or SMY-L/SMY-H solution but the mitoTEMPO group was intraperitoneally treated within mitoTEMPO, a mitochondria targeted antioxidant, for five consecutive days. All the mice except the control group received DOX (10 mg/kg) intraperitoneally on the sixth day, and the mice of the control group were intraperitoneally injected with normal saline at the same time. On the seventh day, all mice were anesthetized and sacrificed, and the heart and serum were collected for further detection.

Adeno-associated virus (AAV) control mice and AAV-Hmox1 overexpress mice were purchased from Obio Technology (Shanghai, China). The mice Hmox1 gene ID was NM_010442. Mice were transfected with the adeno-associated virus at a dose of 9 × 10^11^ viral genome copies for each mouse through injection via the tail vein. Mice were randomly assigned to 4 groups: DOX group, DOX+SMY-H group, DOX+Hmox1-OE group, and DOX+SMY-H+Hmox1-OE group. 8 weeks after transfection, the model induction and drug administration were performed.

### Histopathological examination of heart tissues

Hematoxylin and Eosin (H&E) staining: We fixed the hearts in 4% paraformaldehyde (pH 7.4) overnight, dehydrated them in ethanol, embedded them in paraffin and cut them into 4-μm thick sections. The heart sections were successively dewaxed with xylene, then dehydrated with various concentrations of ethanol. The hematoxylin nuclear staining and eosin cytoplasm staining were conducted. Thereafter, the slides were further dehydrated with diverse concentrations of ethanol, exposed to xylene, and sealed with neutral resin. The histopathological alteration was observed under a light microscope finally.

Sirius red staining: After being fixed in 4% paraformaldehyde over 24 h, the heart tissue was dehydrated in ethanol and embedded in paraffin, then sliced into 4-μm slices using a rotary slicer. After routine dewaxing to water, the slices were stained in Sirius red solution, dehydrated with anhydrous ethanol, put into xylene for hyalinization, and sealed with neutral gum. Finally, the slides were sent for microscopic visualization and analysis.

### Measurement of serum and heart non-heme iron

The mitochondria and cytoplasm of cardiomyocytes were measured individually.

To isolate the mitochondria from cardiomyocytes, a tissue mitochondria isolation kit (C3606, Beyotime Biotechnology) was used in accordance with the manufacturer’s instructions. To obtain serum, blood samples were collected from the orbit of mice and centrifuged at 3000 rpm at 4°C for 10 minutes. Serum, mitochondria, and cytoplasm of cardiomyocytes non-heme iron were measured using the iron assay kit (#ab83366, Abcam). In brief, the collected samples were homogenized using an iron assay buffer. Then iron reducer was added. The supernatant was collected, mixed, and incubated at 37°C for 30 min. Finally, the iron probe was added, mixed, and incubated at 37°C for 1 h protected from light. Consequently, the iron content was immediately measured on a colorimetric microplate reader.

### Measurement of reactive oxygen species

After being harvested, fresh tissues were snap-frozen in liquid nitrogen for the next OCT embedding and sectioning. The slices were washed in PBS buffer to remove OCT glue. DHE probe (UE, D1008) diluted 1:1000 in PBS buffer (final concentration of 5-μM) was added to fully cover the tissue. After incubation at 37°C for 30 min, the sections were washed 1–2 times with PBS to fully remove the DHE which did not enter the tissue. The tablets were sealed with an antifade mounting medium (Beyotime, P0126) and examined under a fluorescence microscope.

### Analysis of mitochondrial membrane potential (ΔΨm)

After being harvested, fresh tissues were snap-frozen in liquid nitrogen before OCT embedding and sectioning. The sections were washed in PBS buffer to remove OCT glue, and stained with JC-1 mitochondrial membrane potential assay kit (Beyotime, C2006) per the manufacturer’s instructions. The sections were sealed with an anti-fluorescence attenuating sealing agent (Beyotime, P0126) and observed using a fluorescence microscope.

### Measurement of MDA, LDH, and CK-MB content

The MDA contents in serum and heart tissues were detected by MDA assay kit (Beyotime, S0131S) respectively. Serum LDH and CK-MB were determined by commercial assay kits (Beyotime, C0016, and ZCIBIO, ZC-38269) in accordance with the manufacturer’s instructions.

### Western blot analysis

Fresh heart tissues were rinsed twice with PBS and then lysed by RIPA lysate containing 1% PMSF and 2% phosphatase inhibitor using a high-throughput tissue homogenizer. After centrifuging the homogenate at 4°C and 12000 rpm for 20 minutes, the supernatant was collected. As directed by the manufacturer, we used a BCA kit to determine the protein concentration in the supernatant. An equal amount of protein was separated by 8–15% SDS-PAGE and transferred onto the PVDF membrane. After incubation and washing with primary and secondary antibodies, the protein bands were exhibited using the Tanon gel imaging system. An analysis of the gray values of the protein bands was conducted using Image J.

### Hmox1 immunofluorescence staining of heart tissue

The heart tissue fixed by 4% paraformaldehyde was dehydrated and then embedded in paraffin. The heart tissue was cut into 4-μm sections using a rotary microtome for later use. Sections were deparaffinized and rehydrated sequentially with xylene and gradient ethanol. Sodium citrate antigen repair solution was used in antigen repair for 10 minutes, and then the slices were put into a 3% hydrogen peroxide (H_2_O_2_) solution and incubated at room temperature for 10 minutes. The slide was blocked by goat serum for 30 minutes, and incubated by the primary antibody (Abcam, ab52947, 1:200 dilution) overnight at 4°C. After discarding the primary antibody, the fluorescent secondary antibody (Abcam, ab150077, diluted 1:1000) was incubated at room temperature for 1 h, and the slides were sealed with neutral balsam. The protein expression was observed under a fluorescence microscope.

### Quantitative real-time PCR of heart tissues

A Trizol (Pufei) solution was used to isolate total RNA from tissues. Spectrophotometry was used to determine RNA concentration and purity. The reverse transcription of RNA was carried out following the manufacturer’s instructions with PrimeScript RT Reagent Kit (Takara, Kusatsu, Japan). An analysis of quantitative PCR was conducted using the CFX96 Real-Time System (Bio-Rad, CA, USA) with SYBR Green Supermix (Bio-Rad) in accordance with the manufacturer's instructions. GAPDH mRNA expression was used as the normalization reference and the 2^−ΔΔCt^ method was used to calculate the fold difference in gene expression. The reactions were performed in triplicate, and melting curve analysis was used to verify specificity. The primers were illustrated in Supplementary Table 1.

### Statistical analysis

Statistical analyses were performed using GraphPad Prism 8.0.2 software. The data are expressed as the means ± standard deviations (SD). An evaluation of statistical significance was performed using a one-way analysis of variance and a two-way analysis of variance. The strategy used for multiple comparisons is Dunnett’s multiple comparisons. *P* < 0.05 as well as *P* < 0.01 were regarded as statistically significant.

## Supplementary Materials

Supplementary Materials

Supplementary Figures

Supplementary Table 1
