# Supplementary material for: Sheng-Mai-Yin inhibits doxorubicin-induced ferroptosis and cardiotoxicity through regulation of Hmox1
Source: Aging (Albany NY). 2023 Sep 28;15(19):10133–45. doi: 10.18632/aging.205062 (PMC10599746; doi:10.18632/aging.205062)
Supplement: Supplementary Materials [file aging-15-205062-s001.pdf]

SUPPLEMENTARY MATERIALS

Supplementary Methods

Echocardiography

On the final day of animal experimentation prior to heart and serum harvesting, echocardiography was performed on mice. The mice were anesthetized with 2% isoflurane and underwent transthoracic echocardiography utilizing the MyLabX5PVET system from Esaote, Italy. Various parameters, including left ventricular internal diameter at end-systole (LVIDs), left ventricular internal diameter at end-diastole (LVIDd), left ventricular end of systole volume (LVESV), and left ventricular end of diastole volume (LVEDV), were obtained and subsequently calculated. The fractional shortening (FS) and ejection fraction (EF) values were determined using the equations FS% = ((LVIDd − LVIDs)/LVIDd) × 100% and EF% = ((LVEDV − LVESV)/LVEDV) × 100%, respectively. The measurement and calculation procedures were conducted by an individual who was blinded to the experimental condition.

Transmission electron microscope (TEM)

After sacrificing the mice, fresh heart tissue blocks (2–3 mm^3) were immediately collected. The samples were fixed overnight in a 2.5% glutaraldehyde solution. The next day, the fixed samples were washed three times with pH 7.0 phosphate buffer (0.1 M) for 15 minutes each. Then, the samples were fixed in a 1% osmium tetroxide solution for 1–2 hours. After washing again with phosphate buffer, the samples were dehydrated using a series of ethanol solutions (30%, 50%, 70%, 80%, 90%, and 95%) for 15 minutes each, followed by a 20-minute treatment with 100% ethanol. Subsequently, the samples were treated with pure acetone for 20 minutes. The samples were embedded in a mixture of embedding medium and acetone (1:1) for 1 hour, followed by a mixture of embedding medium and acetone (3:1) for 3 hours. After embedding in pure embedding medium overnight, the samples were heated at 70°C. Ultra-thin sections (70–90 nm) were obtained using an ultramicrotome. The sections were stained with lead citrate and uranyl acetate-ethanol solution (50% saturation) for 5–10 minutes each, and then air-dried. Finally, the sections were observed under a transmission electron microscope (Hitachi H-7650).

Detection of glutathione (GSH)

The freshly acquired hearts were rapidly frozen in liquid nitrogen, pulverized into a fine powder, and subsequently subjected to GSH activity analysis utilizing a GSH kit (S0053, Beyotime).

Supplementary Results

Sheng-Mai-Yin restores heart function via Hmox1

In order to comprehensively assess the impact of Sheng-Mai-Yin (SMY) on impaired cardiac function, we performed echocardiography to obtain crucial measurements such as fractional shortening (FS) and ejection fraction (EF) (Supplementary Figure 1A). These parameters serve as key indicators of cardiac contractility and overall heart function. Our findings revealed significant improvements in heart function following SMY treatment. Both the SMY-H (high-dose SMY) and mitoTEMPO groups exhibited preserved cardiac function compared to the control group (Supplementary Figure 1B, 1C). Specifically, the FS and EF values were markedly higher in the SMY-H and mitoTEMPO groups, indicating enhanced systolic function and better overall pump efficiency. These results suggest that SMY and mitoTEMPO have cardioprotective effects, leading to the preservation or restoration of heart function. However, it is noteworthy to highlight the impact of Hmox1 overexpression on the cardioprotective effects of SMY (Supplementary Figure 1B, 1C). Despite the positive outcomes observed in the SMY-H group, the beneficial effects were attenuated when Hmox1 was overexpressed. This finding suggests a potential interaction or interference between SMY and Hmox1 signaling pathways, which requires further investigation.

Observation of mitochondrial ultrastructure in heart tissues

The control group's mitochondria displayed conventional ultrastructural characteristics, including undamaged double membranes, clearly defined cristae, and a compact matrix. These mitochondria exhibited elongated shapes and uniform distribution within the cytoplasm, signifying a typical mitochondrial morphology and functionality. The group treated with DOX displayed noteworthy changes in mitochondrial structure, as evidenced by TEM analysis. Specifically, the mitochondria exhibited marked swelling, accompanied by an enlargement in size and a loss of the characteristic cristae structure. These findings suggest that DOX-induced mitochondrial damage resulted in mitochondrial dysfunction and impaired energy production. Notably, treatment with SMY or mitoTEMPO effectively preserved mitochondrial integrity in the face of DOX-induced damage. However, the protective effects of SMY on mitochondrial morphology were negated by the overexpression of Hmox1 (Supplementary Figure 2).

Sheng-Mai-Yin restores GSH levels in DOX treated mice

The results of the experiment demonstrate a significant reduction in glutathione (GSH) levels in the hearts of mice treated with DOX, as depicted in Supplementary Figure 3. Conversely, SMY treatment exhibited a dose-dependent restoration of GSH levels, comparable to the effects of mitoTEMPO, thereby indicating its potential to enhance resistance against oxidative damage.
