# Supplementary material for: Sheng-Mai-Yin inhibits doxorubicin-induced ferroptosis and cardiotoxicity through regulation of Hmox1
Source: Aging (Albany NY). 2023 Sep 28;15(19):10133–45. doi: 10.18632/aging.205062 (PMC10599746; doi:10.18632/aging.205062)
Supplement: Supplementary Figures [file aging-15-205062-s002.pdf]

SUPPLEMENTARY FIGURES

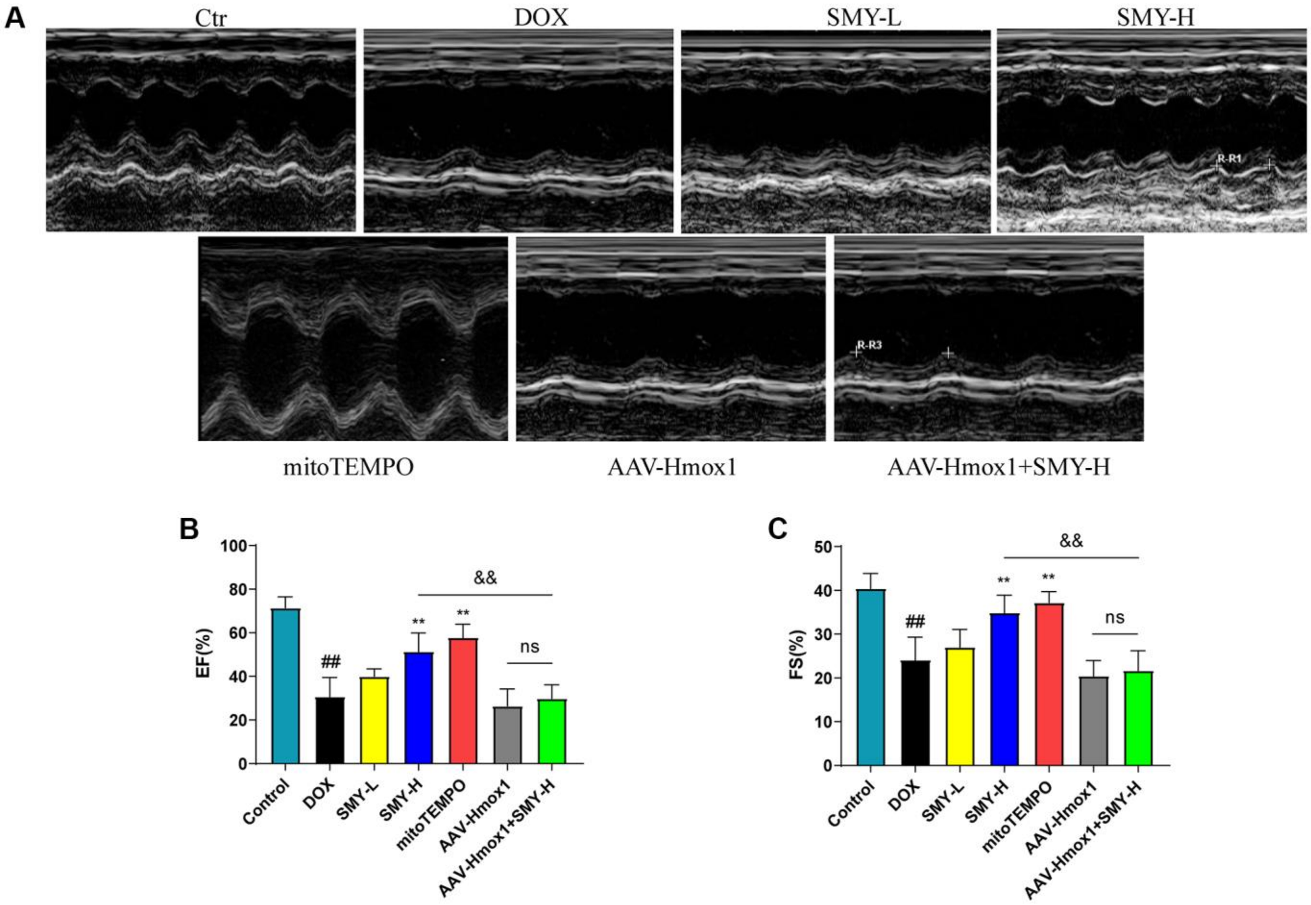

Supplementary Figure 1. Echocardiography analysis for evaluating cardiac function. (**A**) Representative images of echocardiography. (**B**) EF values of different groups. (**C**) FS values of different groups. The results were presented as mean ± SEM. ##means compared with control group, *P* < 0.01; *means compared with DOX group, *P* < 0.05, **means compared with DOX group, *P* < 0.01, &&means between the indicated groups, *P* < 0.01, ns means no significance between the indicated groups.

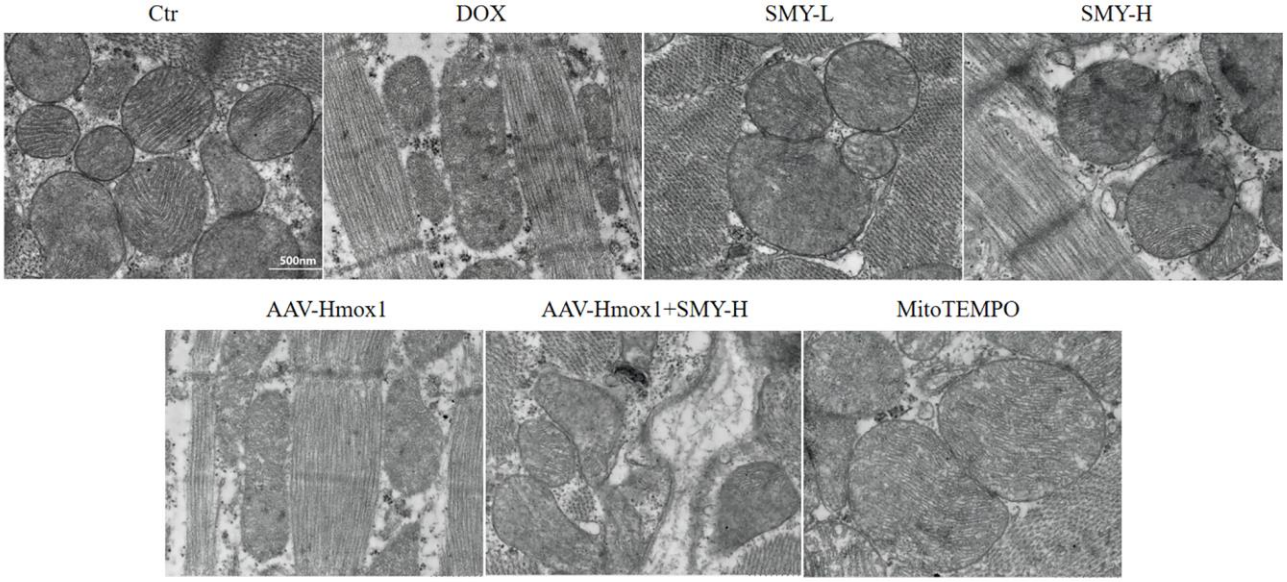

Supplementary Figure 2. Representative mitochondrial TEM images of heart tissues in different groups.

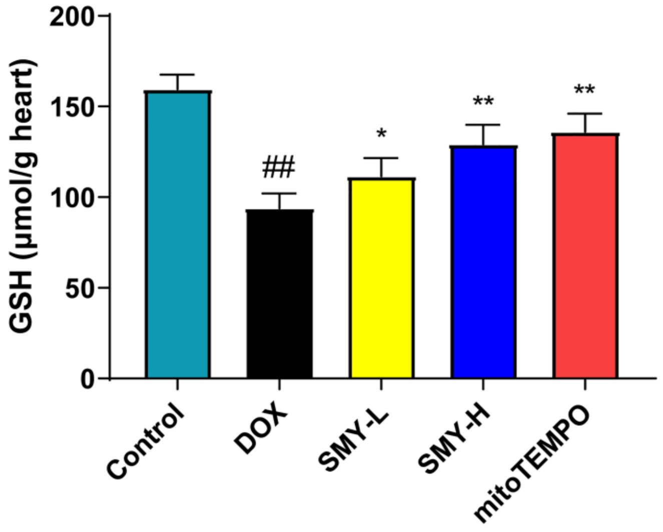

Supplementary Figure 3. GSH levels detected by commercial kits in heart tissues.
