# Supplementary material for: Sheng-Mai-Yin inhibits doxorubicin-induced ferroptosis and cardiotoxicity through regulation of Hmox1
Source: Aging (Albany NY). 2023 Sep 28;15(19):10133–45. doi: 10.18632/aging.205062 (PMC10599746; doi:10.18632/aging.205062)
Supplement: Supplementary Table 1 [file aging-15-205062-s003.pdf]

SUPPLEMENTARY TABLE

Supplementary Table 1. The primers used for quantitative PCR.

	Forward primer (5′→3′)	Reverse primer (5′→3′)
Ptgs2	CTGCGCCTTTTCAAGGATGG	GGGGATACACCTCTCCACCA
Hmox1	GGTGATGGCTTCCTTGTACC	AGTGAGGCCCATACCAGAAG
GAPDH	ATCATCCCTGCATCCAC	ATCCACGACGGACACATT
